# Supplementary figures and images for: BMP2 alterations in mucinous cystadenocarcinoma of the breast: insights from whole-exome sequencing
Source: PeerJ. 2025 Sep 3;13:e19948. doi: 10.7717/peerj.19948 (PMC12422278; doi:10.7717/peerj.19948)

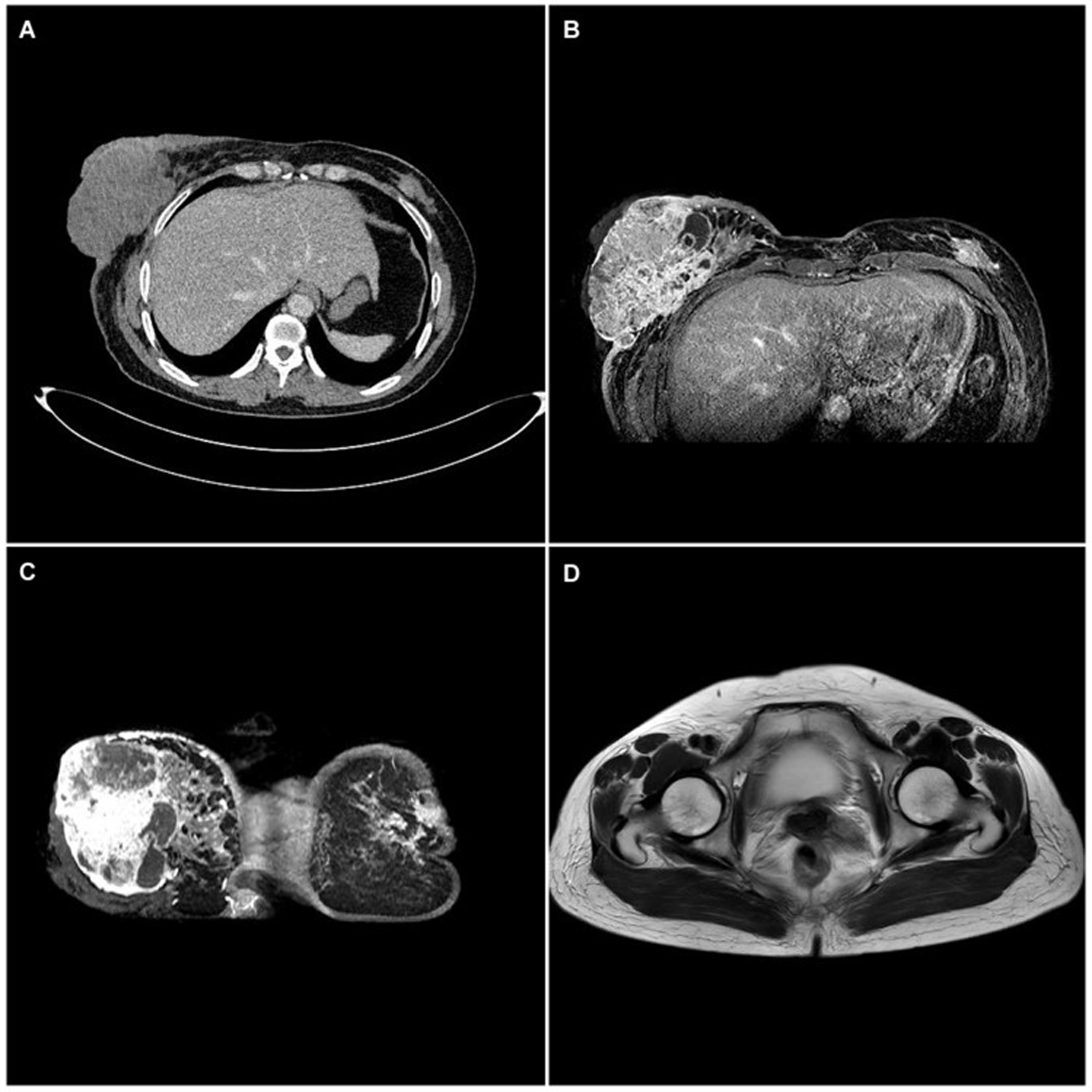

Supplement: Supplemental Information 1 — (B-C) A large mass in the outer part of the right breast, and invaded 381 the chest wall and skin, and a mass of the left breast in MRI in case 1. (D) The pelvic MRI of case 1. [file peerj-13-19948-s001.jpg]

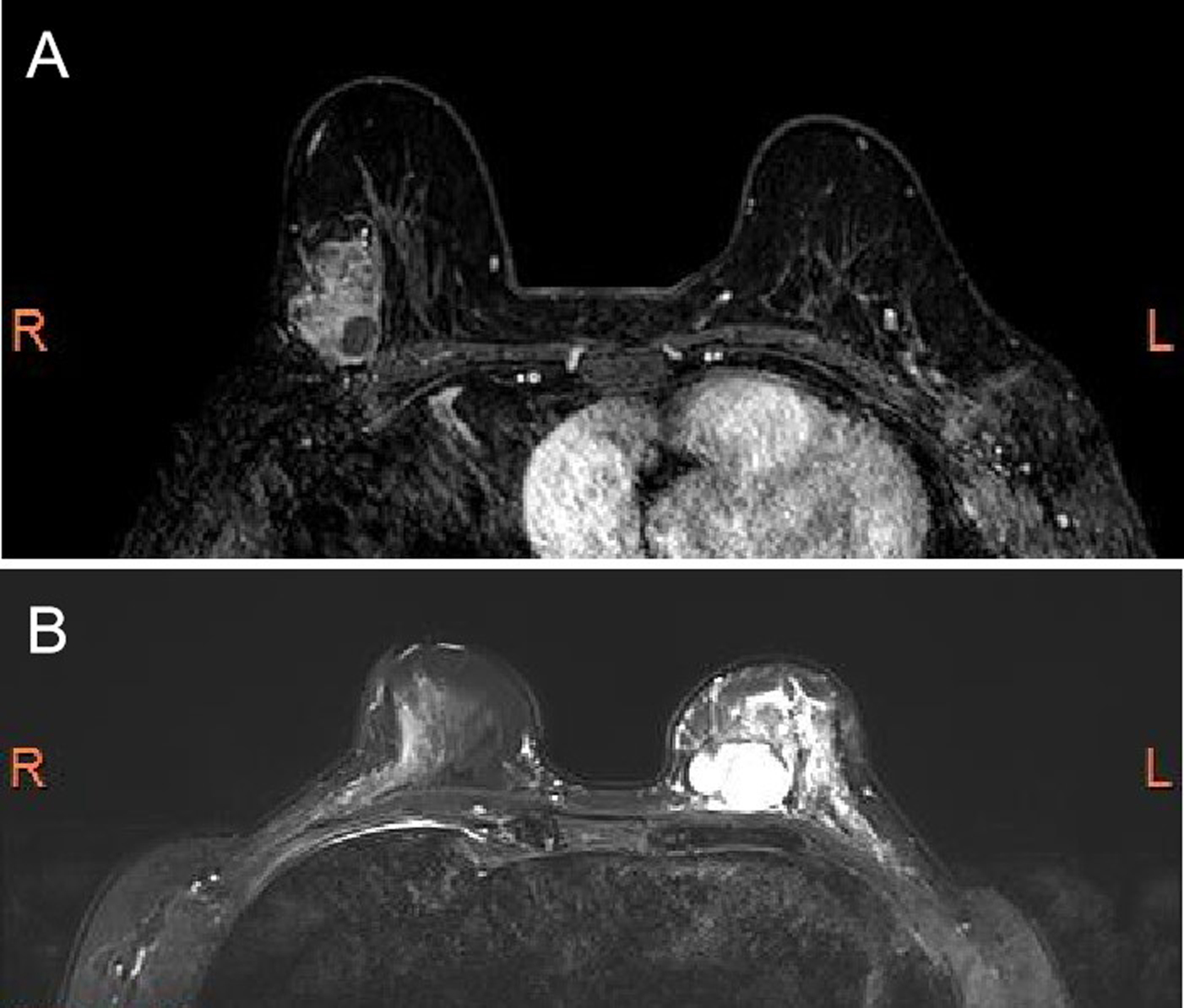

Supplement: Supplemental Information 2 [file peerj-13-19948-s002.jpg]

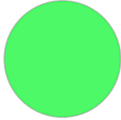

**U2-type catalytic  
step 2 spliceosome**

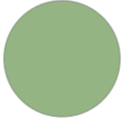

**intrinsic component  
of external side of  
plasma membrane**

Supplement: Supplemental Information 3 [file peerj-13-19948-s003.zip › BMP2/BMP2/CC.pdf]

# American Joint Committee on Cancer Metastasis Stage Code

# samples (%)

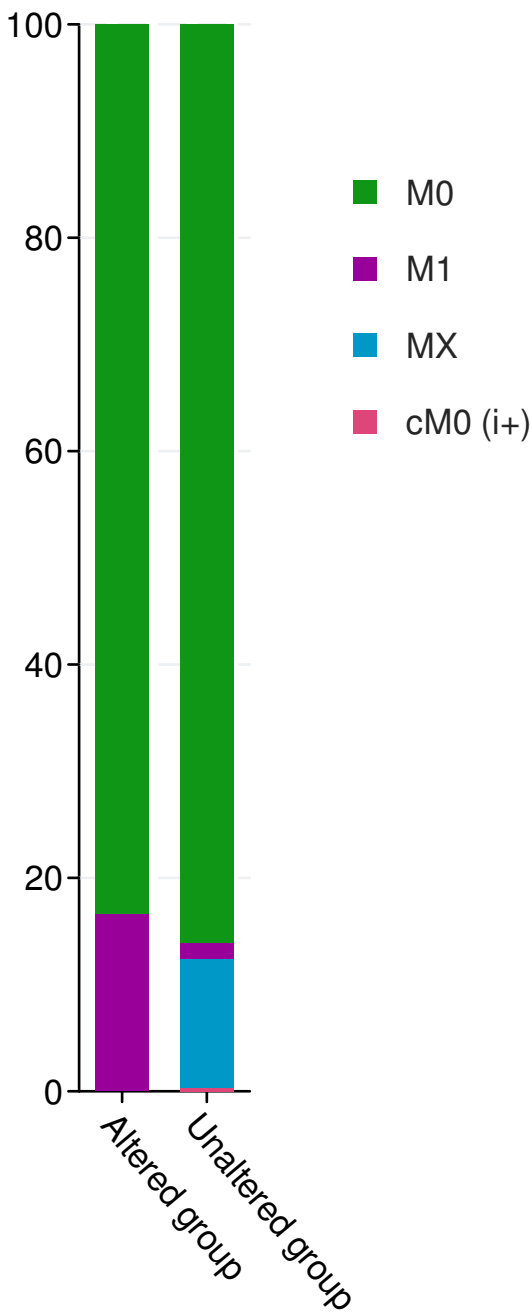

Group

Supplement: Supplemental Information 3 [file peerj-13-19948-s003.zip › BMP2/BMP2/clinical-plot-svg metastatic TCGA.pdf]

Pam50 + Claudin-low subtype  
# samples (%)

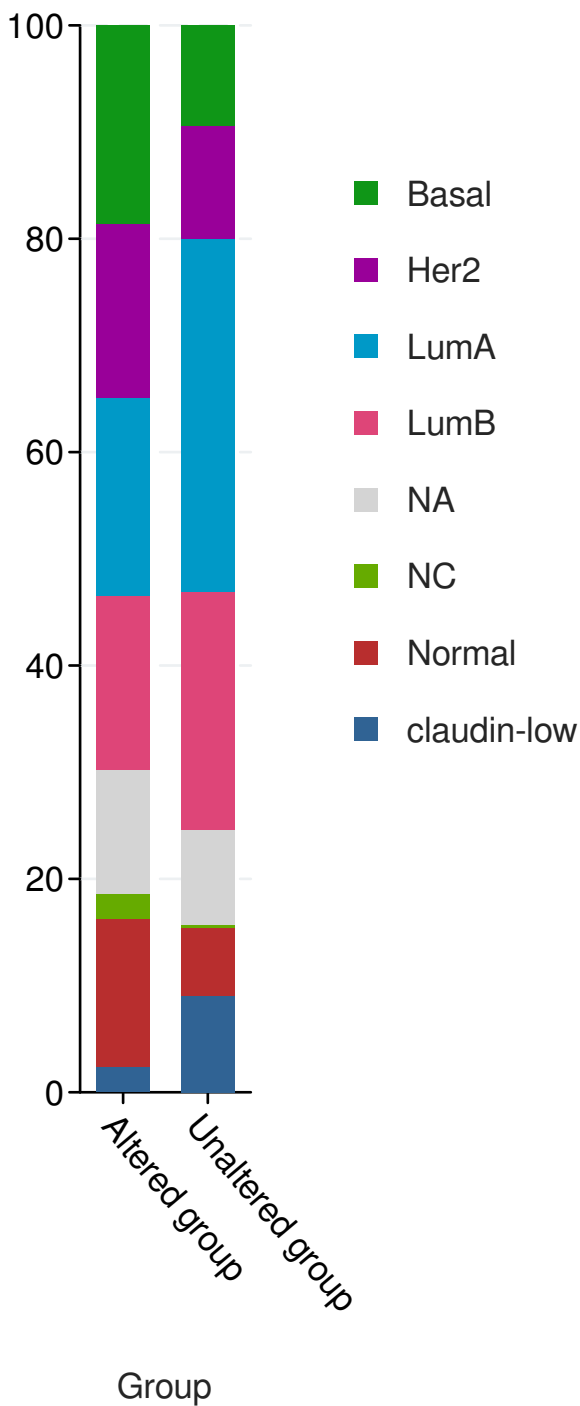

Supplement: Supplemental Information 3 [file peerj-13-19948-s003.zip › BMP2/BMP2/clinical-plot-svg PAM50 METABRIC.pdf]

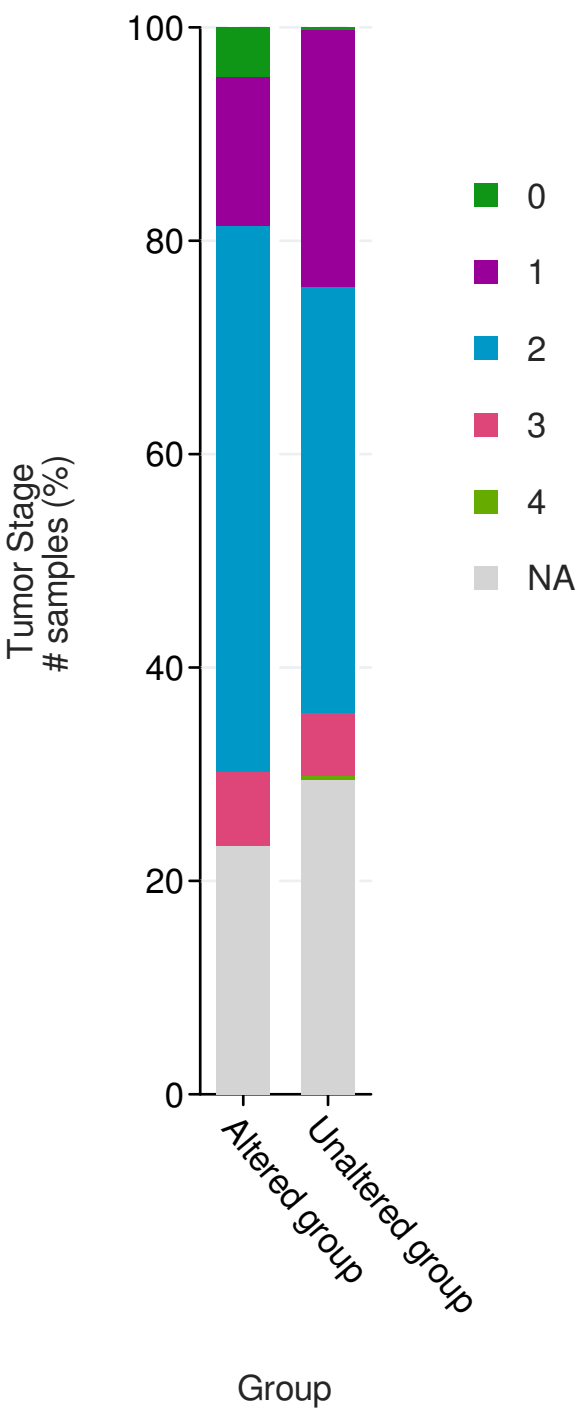

Supplement: Supplemental Information 3 [file peerj-13-19948-s003.zip › BMP2/BMP2/clinical-plot-svg tumor stage METABRIC.pdf]

Metastatic tumor indicator  
# samples (%)

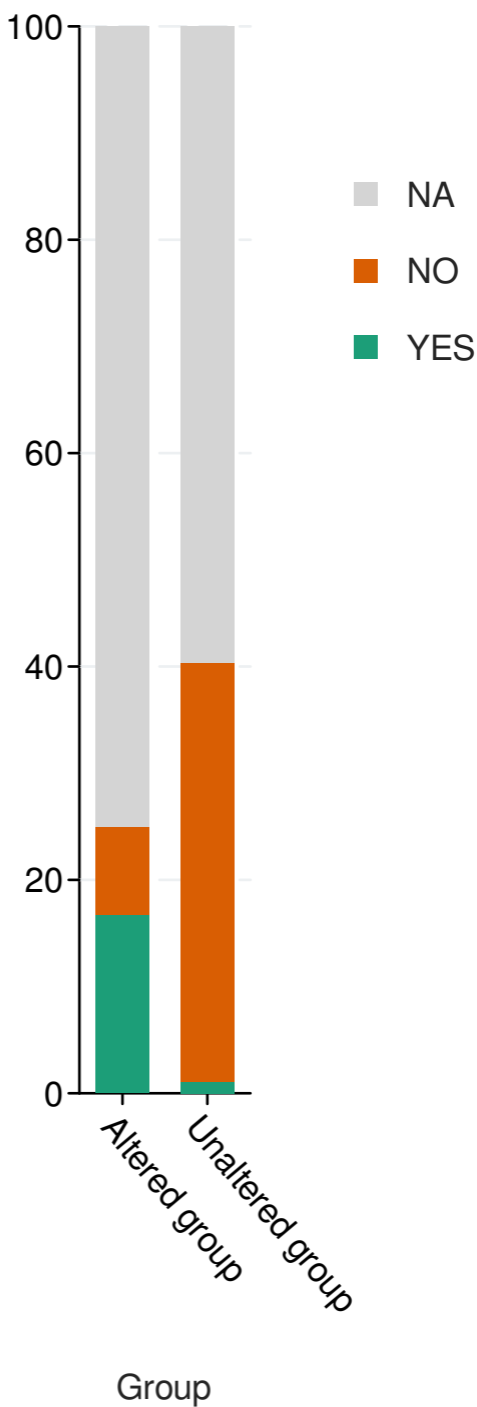

Supplement: Supplemental Information 3 [file peerj-13-19948-s003.zip › BMP2/BMP2/clinical-plot-svgMETASTATIC TCGA.pdf]

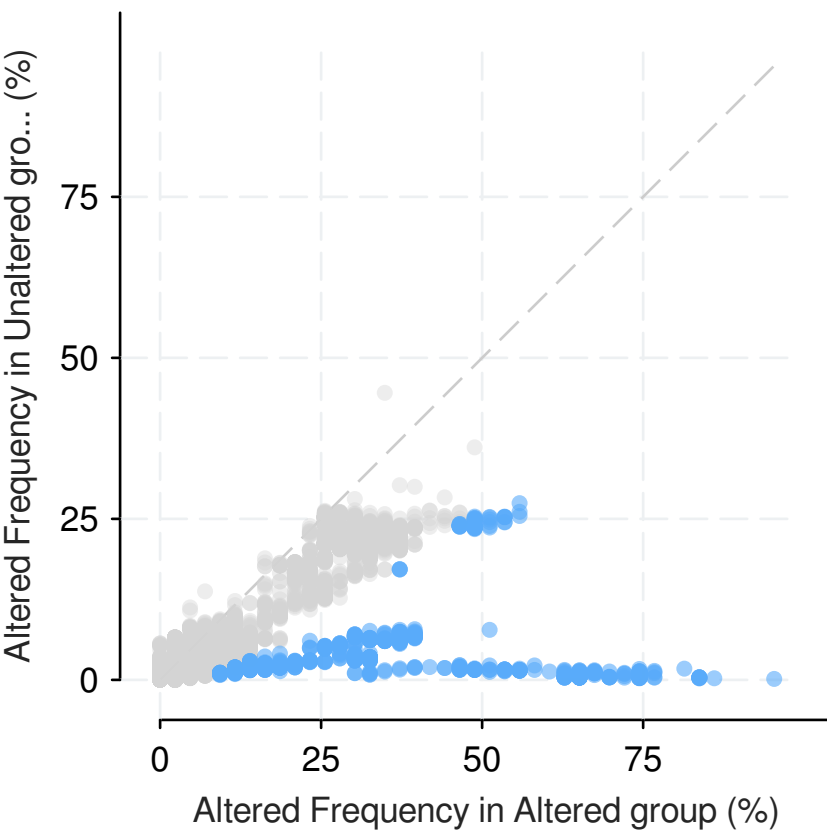

Supplement: Supplemental Information 3 [file peerj-13-19948-s003.zip › BMP2/BMP2/enrichments-frequency-scatter_METABRIC.pdf]

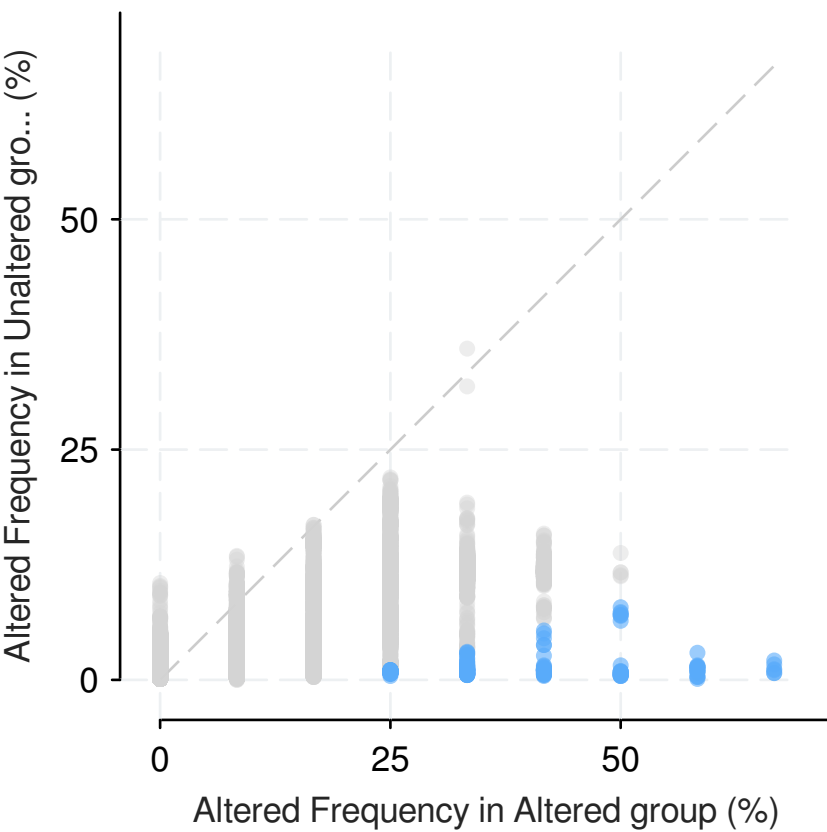

Supplement: Supplemental Information 3 [file peerj-13-19948-s003.zip › BMP2/BMP2/enrichments-frequency-scatterTCGA.pdf]

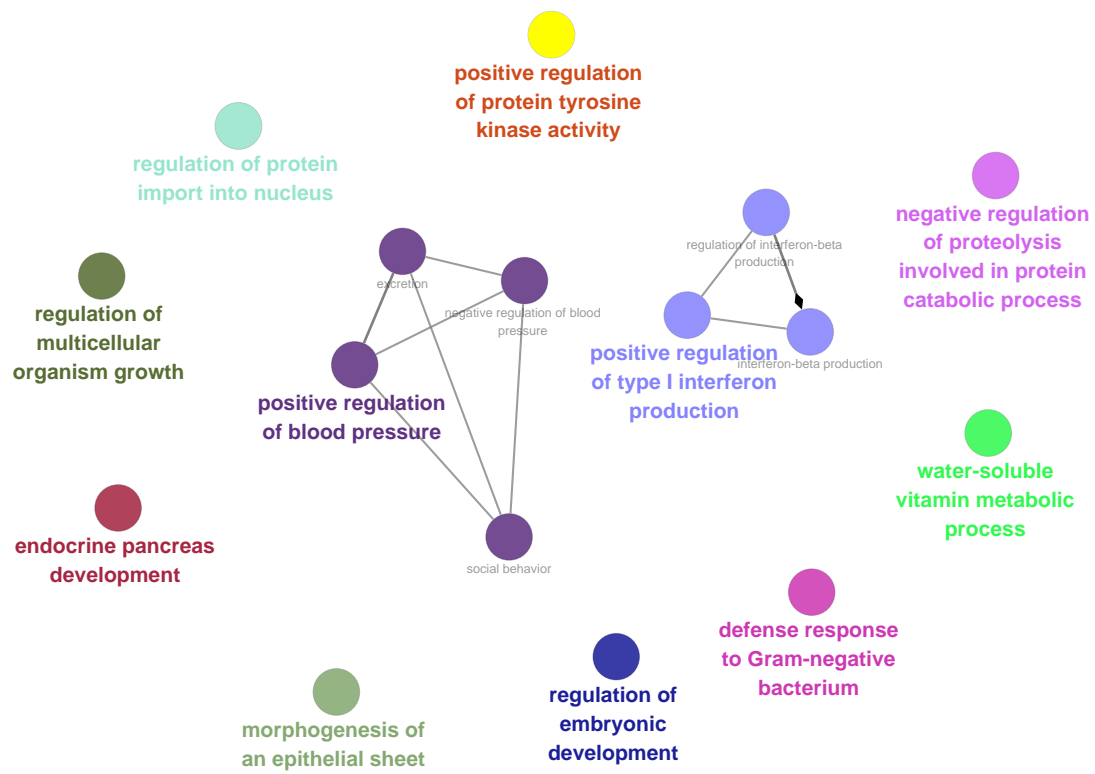

Supplement: Supplemental Information 3 [file peerj-13-19948-s003.zip › BMP2/BMP2/GO BP.pdf]

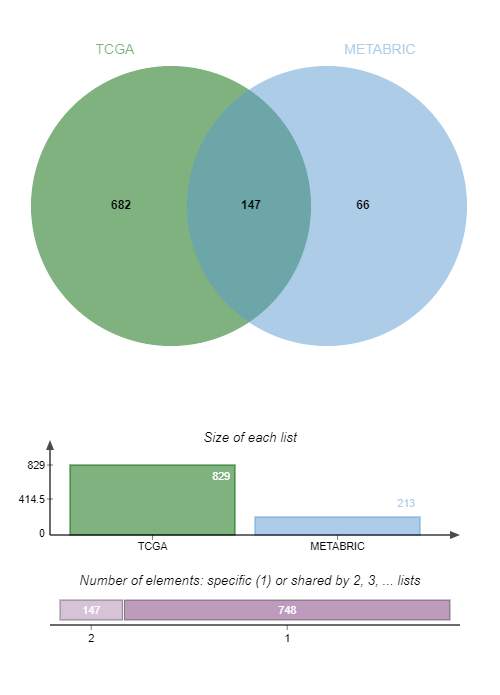

Supplement: Supplemental Information 3 [file peerj-13-19948-s003.zip › BMP2/BMP2/jVenn_chart.png]

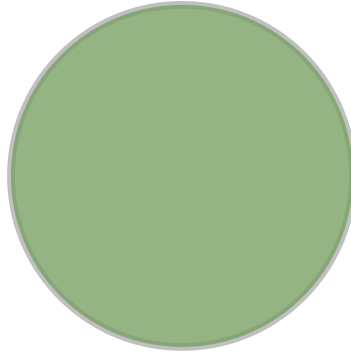

# GnRH secretion

Supplement: Supplemental Information 3 [file peerj-13-19948-s003.zip › BMP2/BMP2/KEGG.pdf]

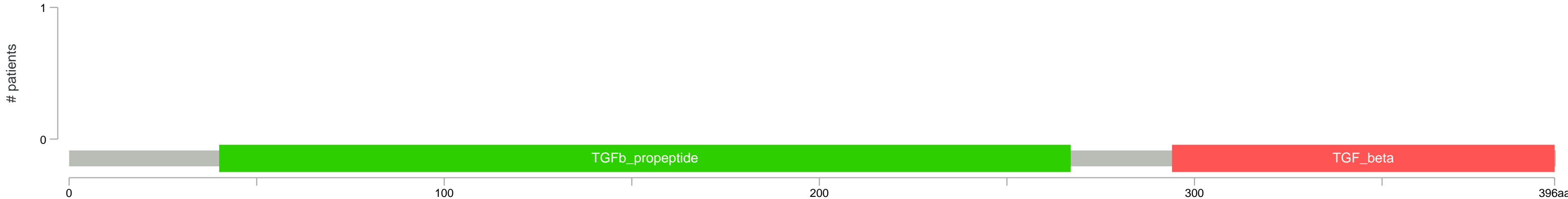

Supplement: Supplemental Information 3 [file peerj-13-19948-s003.zip › BMP2/BMP2/METABRIC BMP2_lollipop.pdf]

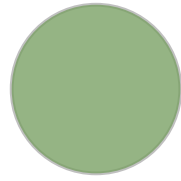

**positive regulation  
of protein tyrosine  
kinase activity**

Supplement: Supplemental Information 3 [file peerj-13-19948-s003.zip › BMP2/BMP2/MF.pdf]

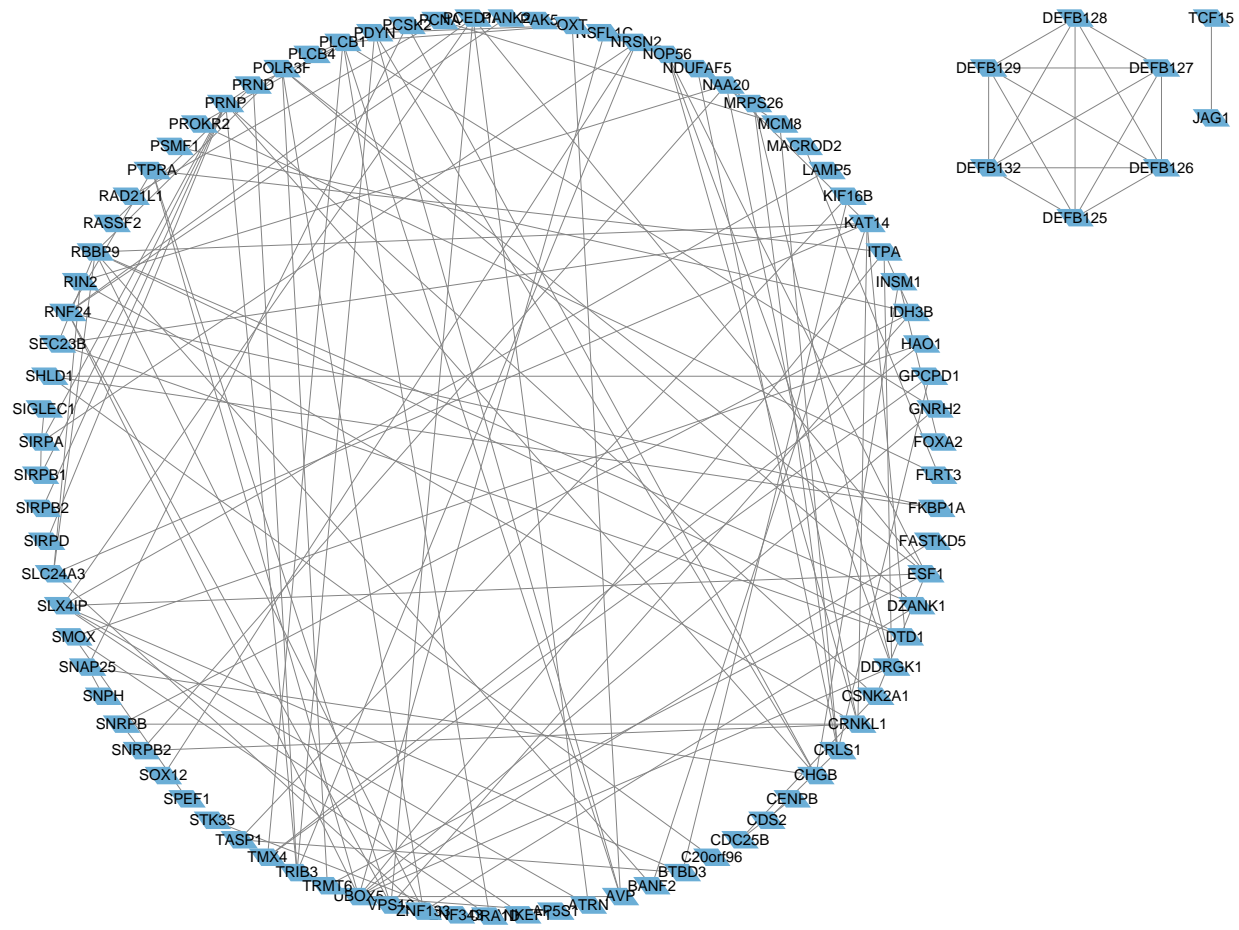

Supplement: Supplemental Information 3 [file peerj-13-19948-s003.zip › BMP2/BMP2/net.network.txt.pdf]

Logrank Test P-Value: 0.0654

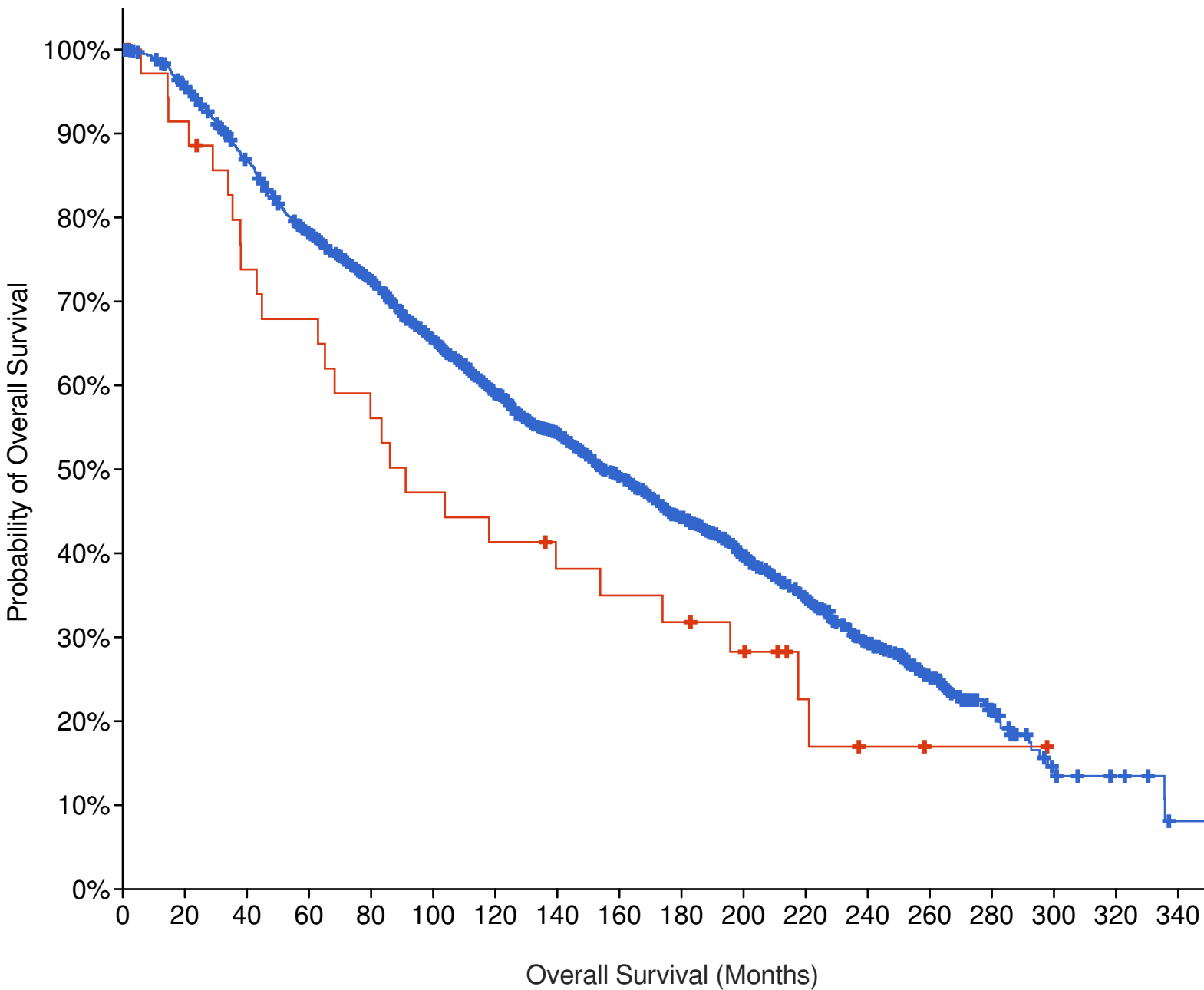

Overall

- Altered group
- Unaltered group

Supplement: Supplemental Information 3 [file peerj-13-19948-s003.zip › BMP2/BMP2/Overall_ METABRIC.pdf]

Logrank Test P-Value: 6.259e-3

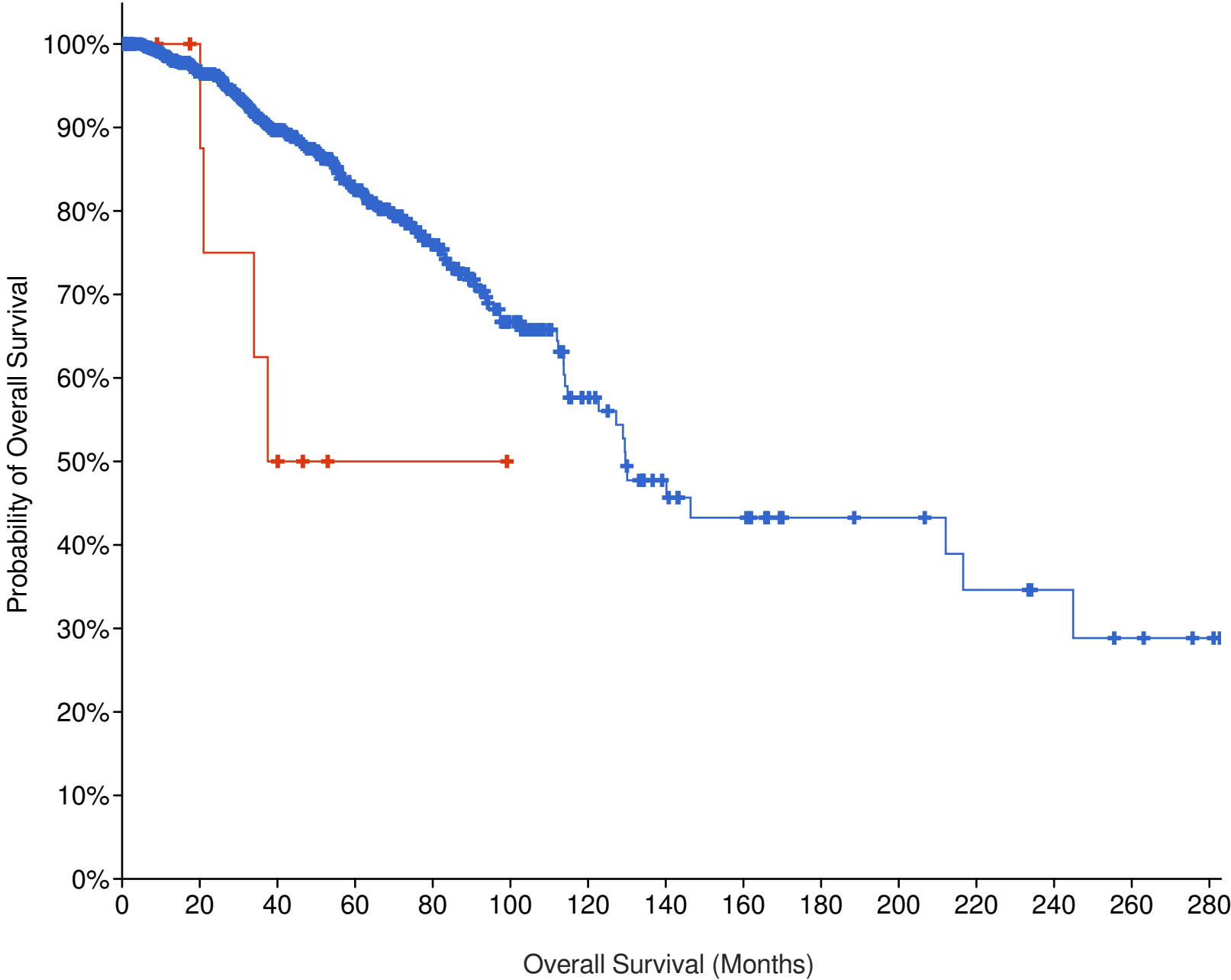

Overall

- Altered group
- Unaltered group

Supplement: Supplemental Information 3 [file peerj-13-19948-s003.zip › BMP2/BMP2/Overall_ TCGA.pdf]

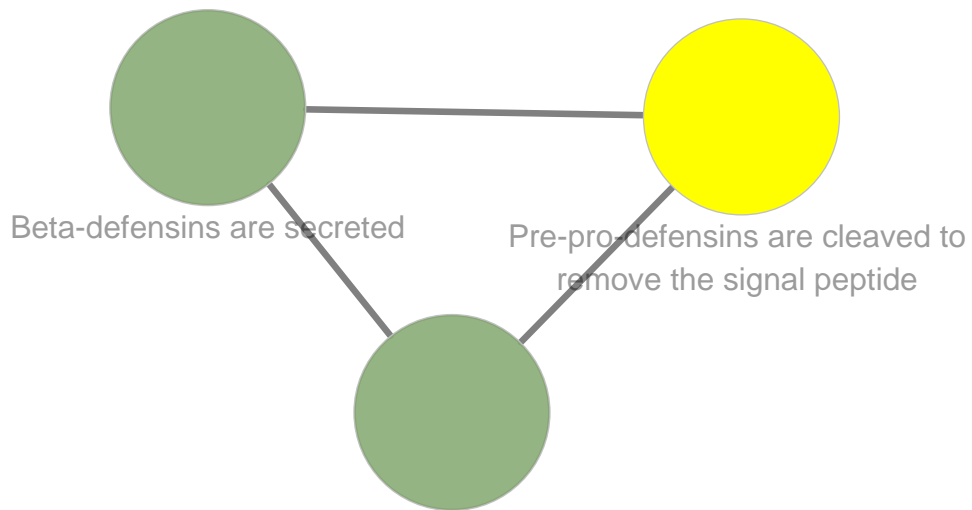

**Beta-defensins bind  
microbial  
membranes causing  
disruption**

Supplement: Supplemental Information 3 [file peerj-13-19948-s003.zip › BMP2/BMP2/REACTOME.pdf]

Logrank Test P-Value: 3.218e-3

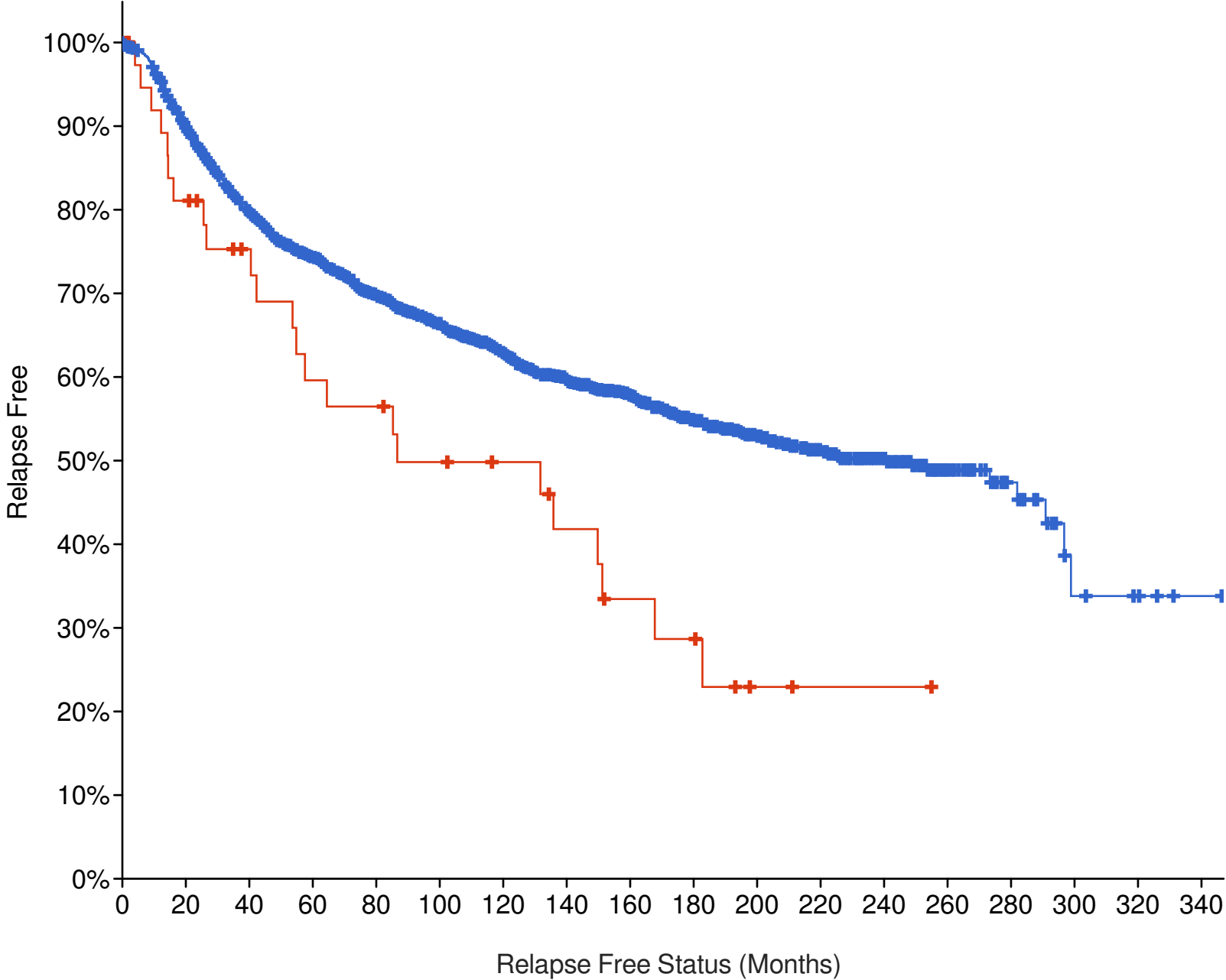

**Relapse Free**

- Altered group
- Unaltered group

Supplement: Supplemental Information 3 [file peerj-13-19948-s003.zip › BMP2/BMP2/Relapse_Free METABRIC.pdf]

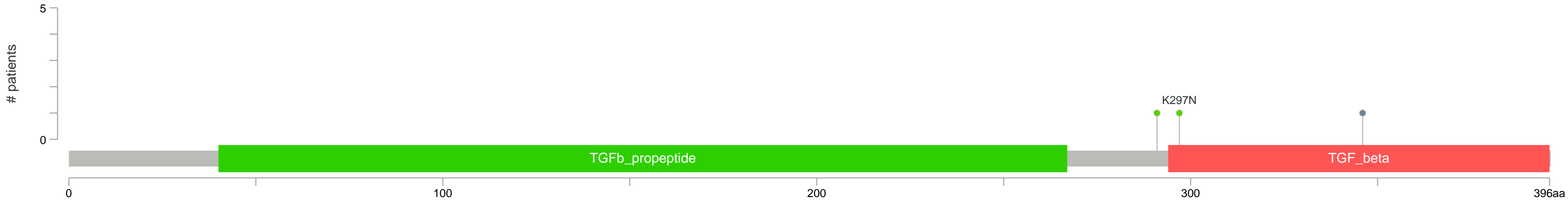

Supplement: Supplemental Information 3 [file peerj-13-19948-s003.zip › BMP2/BMP2/TCGA BMP2_lollipop.pdf]

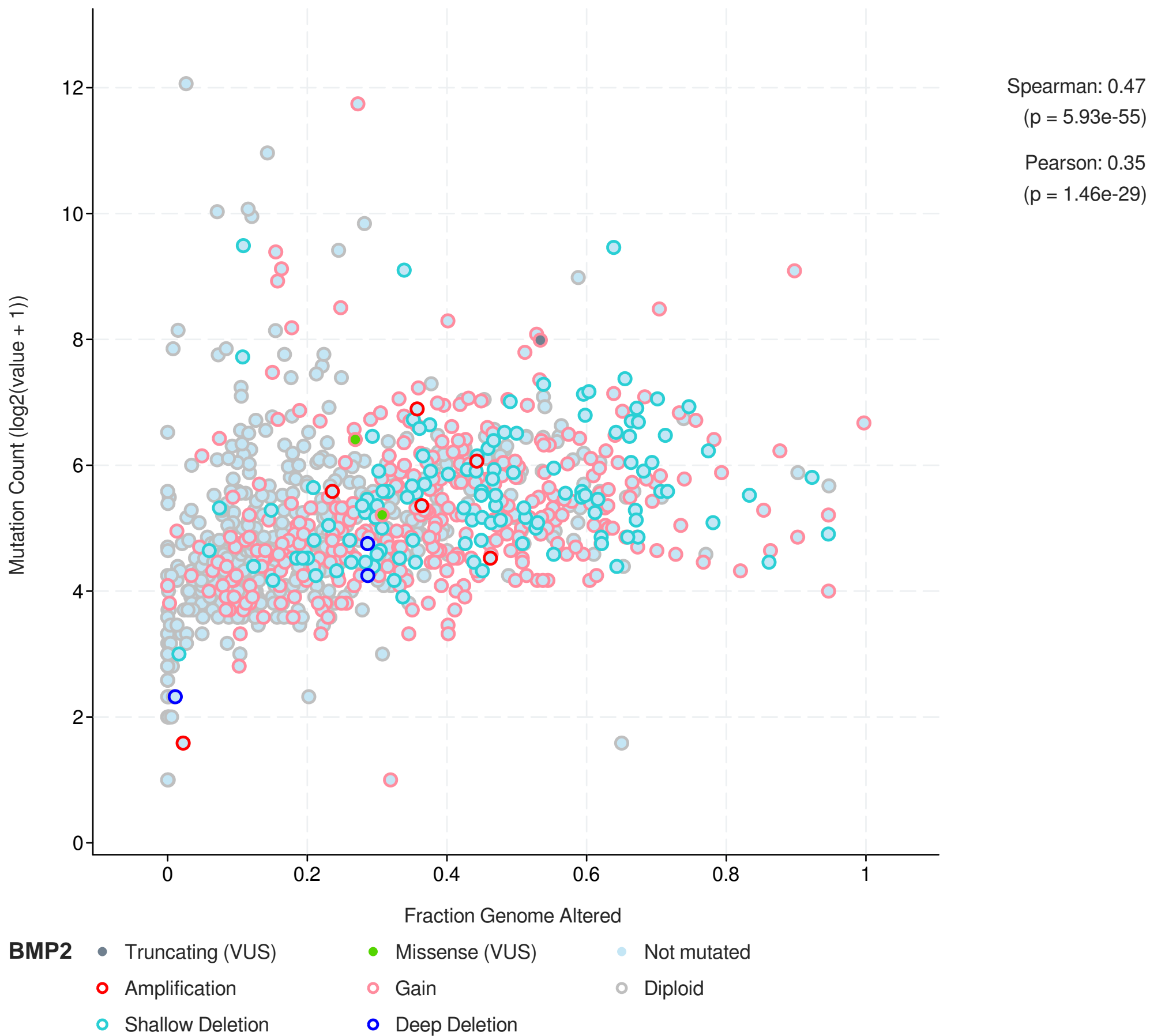

Supplement: Supplemental Information 3 [file peerj-13-19948-s003.zip › BMP2/BMP2/TCGA plot FGA BMP2.pdf]

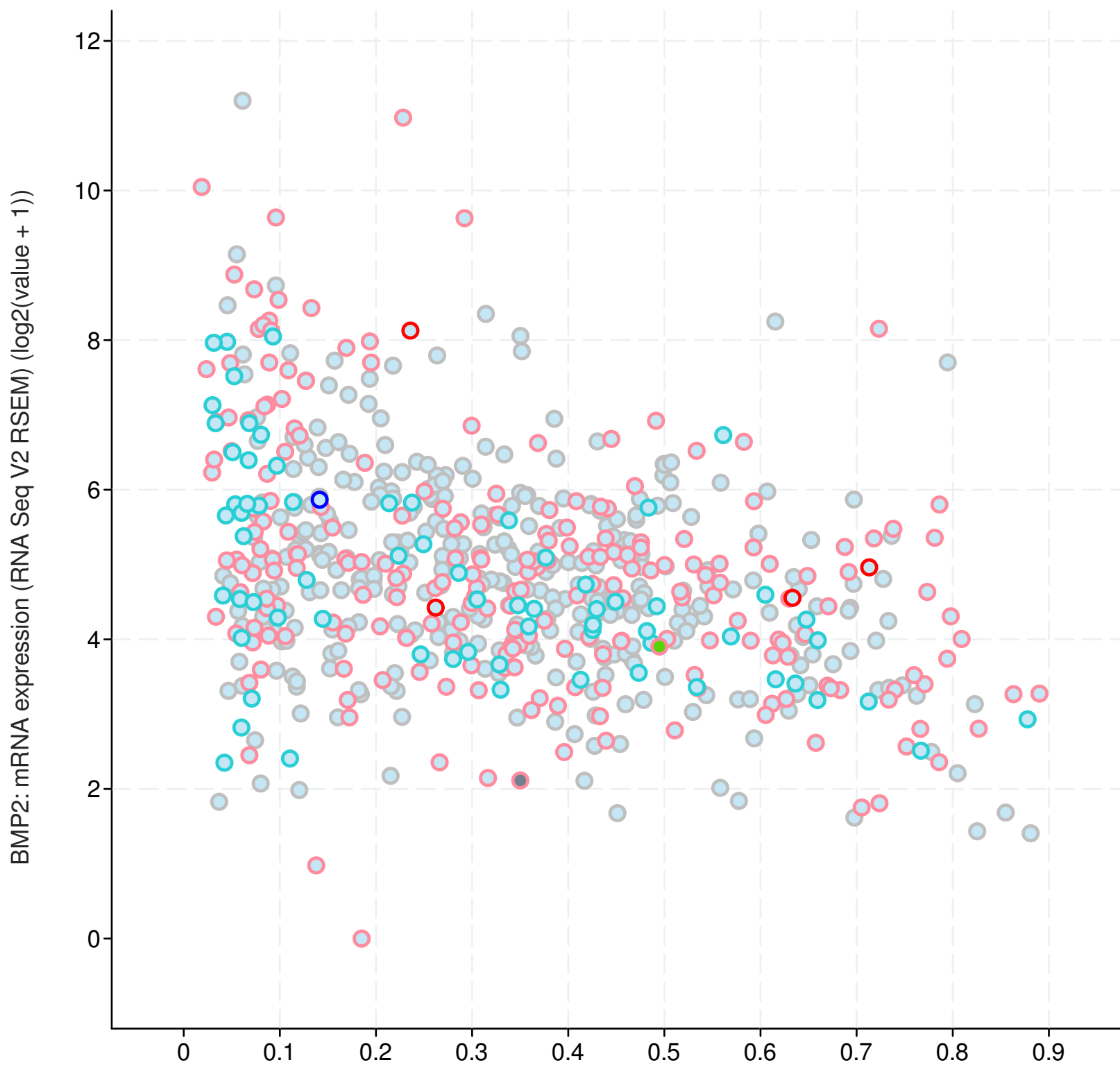

Spearman: -0.35  
( $p = 1.77\text{e-}20$ )

Pearson: -0.37  
( $p = 6.73\text{e-}23$ )

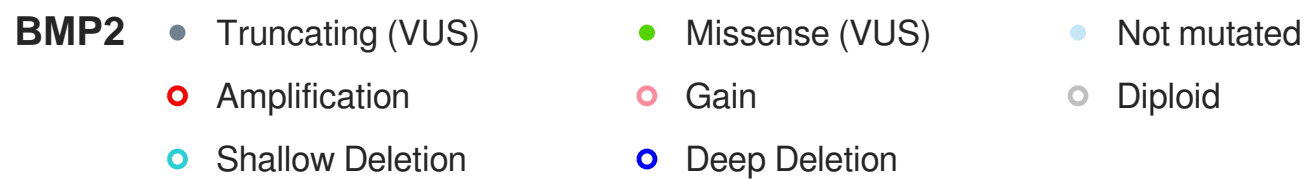

Supplement: Supplemental Information 3 [file peerj-13-19948-s003.zip › BMP2/BMP2/TCGA plot Methylation BMP2.pdf]

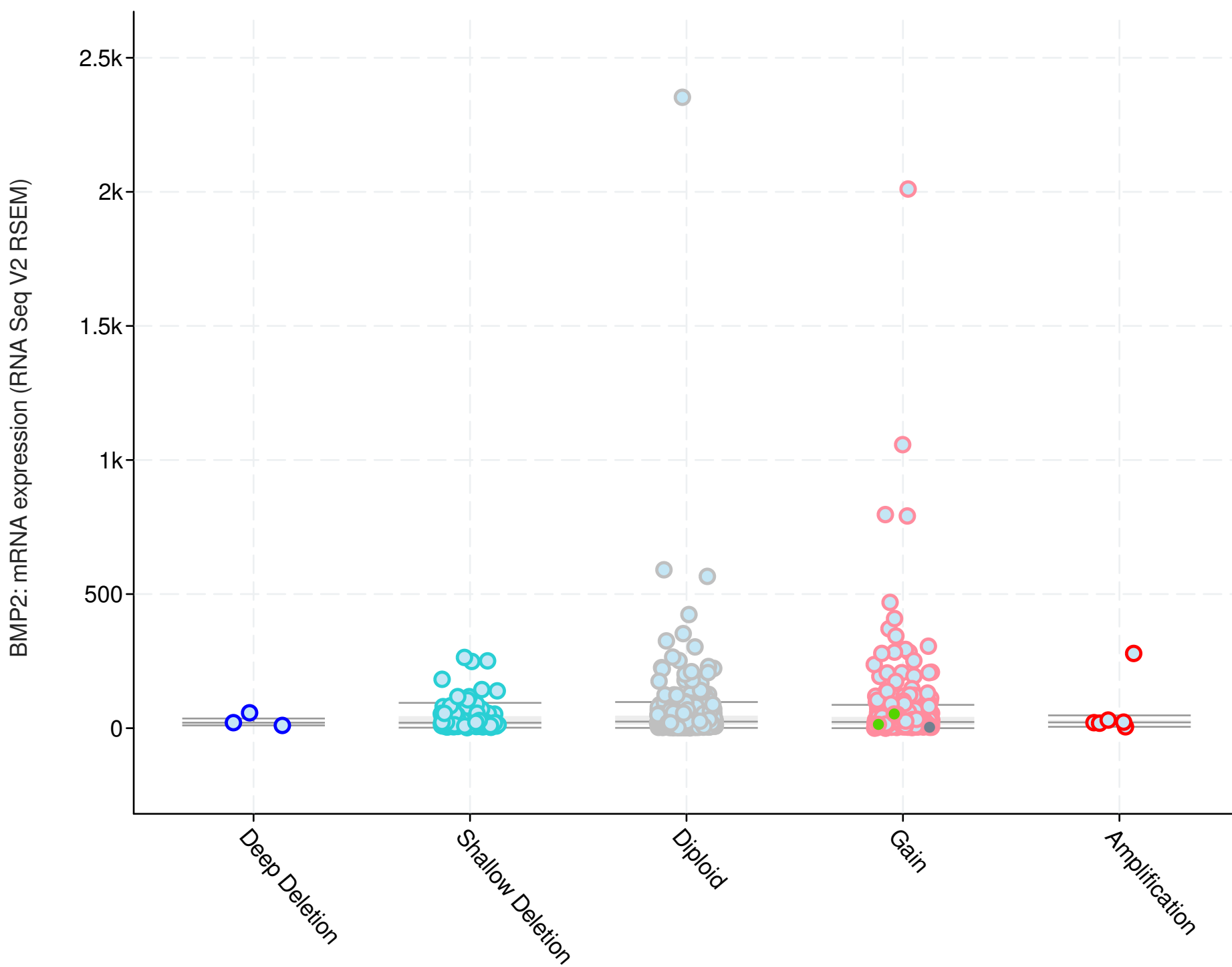

BMP2: Putative copy-number alterations from GISTIC

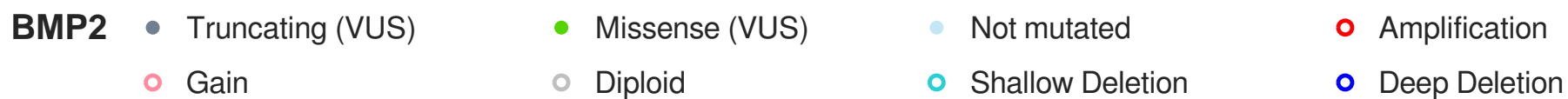

Supplement: Supplemental Information 3 [file peerj-13-19948-s003.zip › BMP2/BMP2/TCGAplot BMP2.pdf]

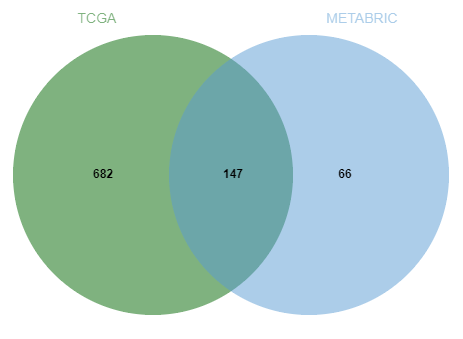

Supplement: Supplemental Information 3 [file peerj-13-19948-s003.zip › BMP2/BMP2/venn.tif]

# Altered in 3 (100%) of 3 samples.

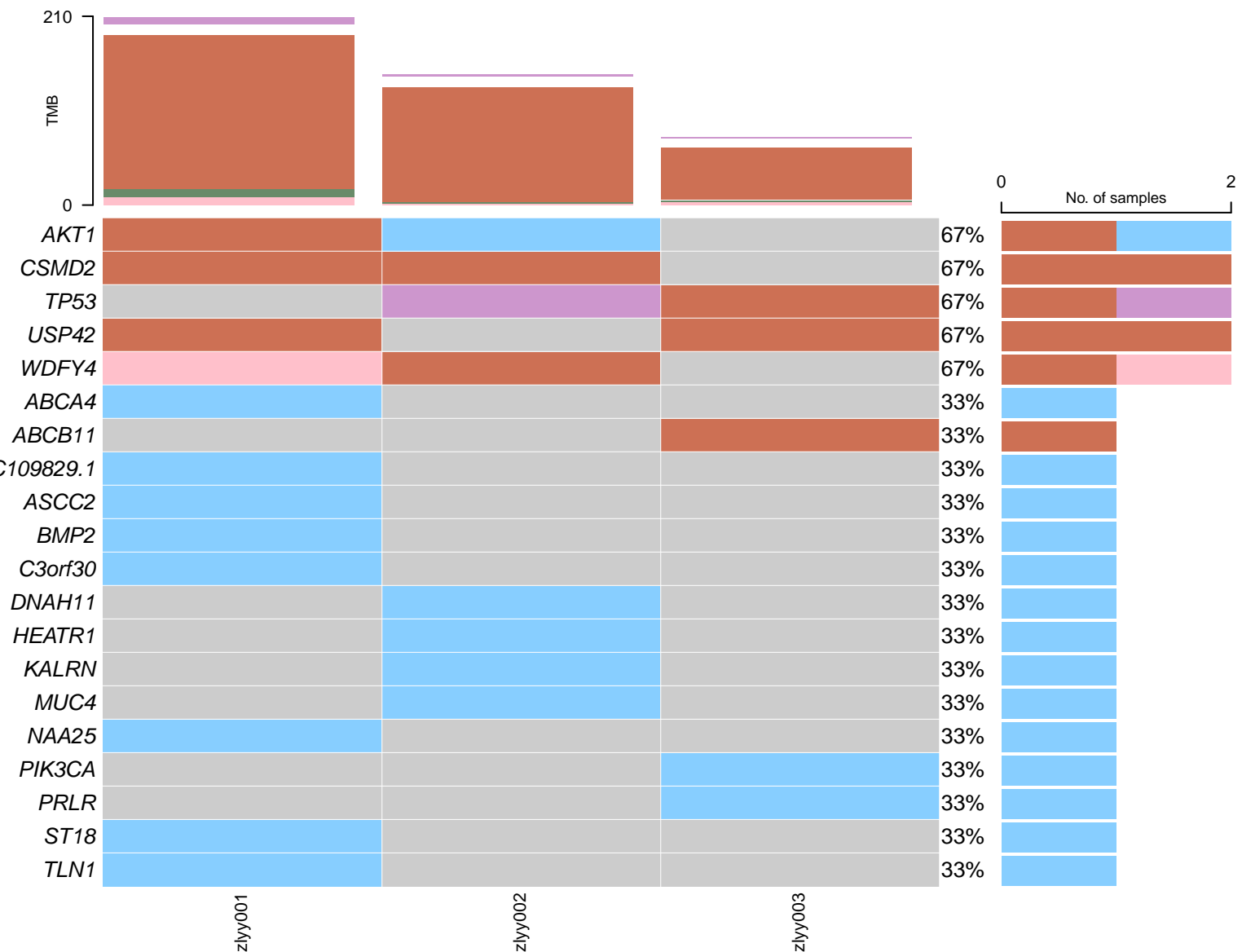

Supplement: Supplemental Information 3 [file peerj-13-19948-s003.zip › TK-CQ-2023-0609-004/1.Ocnoplot═╝/oncoplot.pdf]

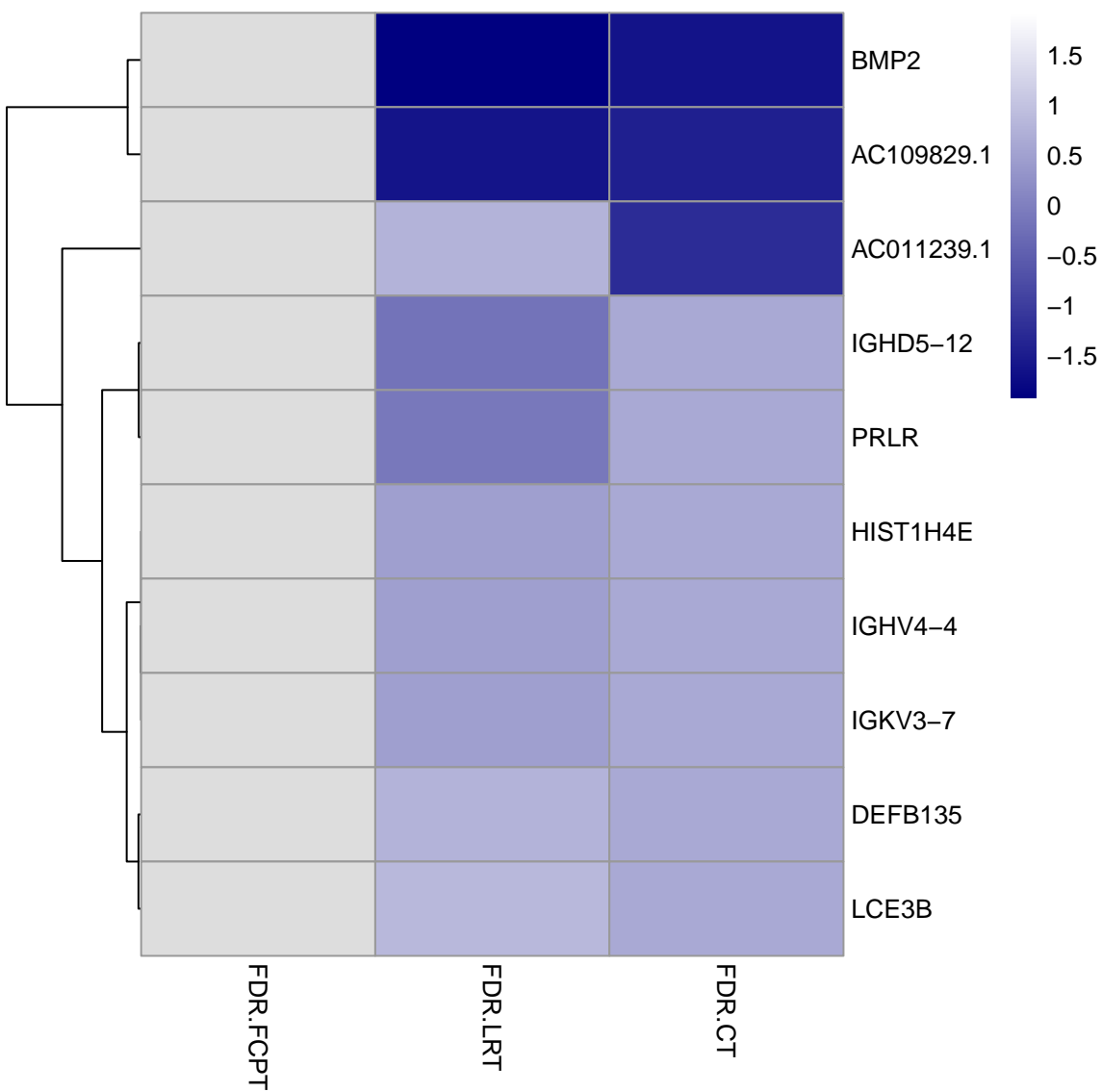

Supplement: Supplemental Information 3 [file peerj-13-19948-s003.zip › TK-CQ-2023-0609-004/2.╧╘╓°═╗▒Σ╗∙╥≥/smgs_FDR.pdf]

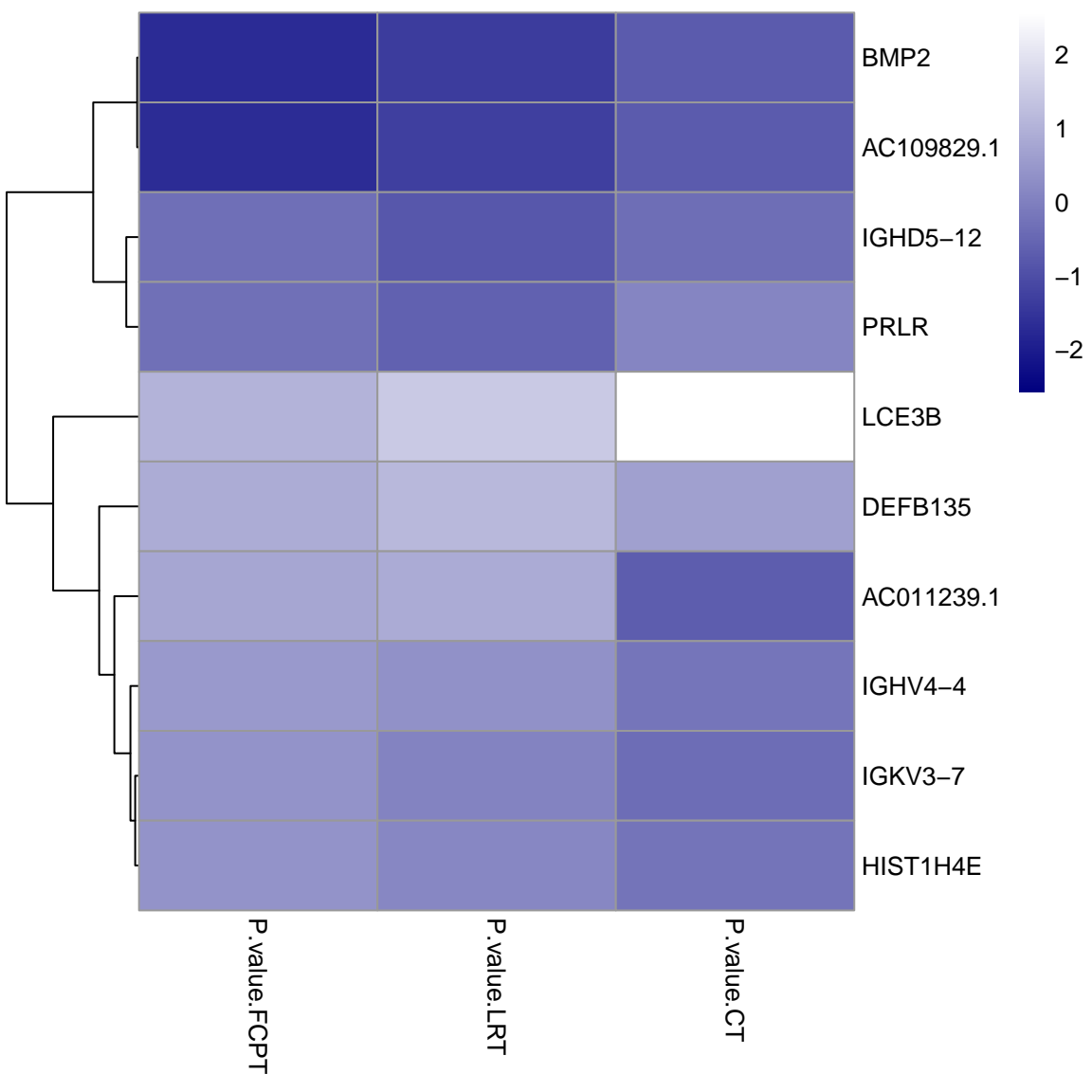

Supplement: Supplemental Information 3 [file peerj-13-19948-s003.zip › TK-CQ-2023-0609-004/2.╧╘╓°═╗▒Σ╗∙╥≥/smgs_pvalue.pdf]

*zlyy001*

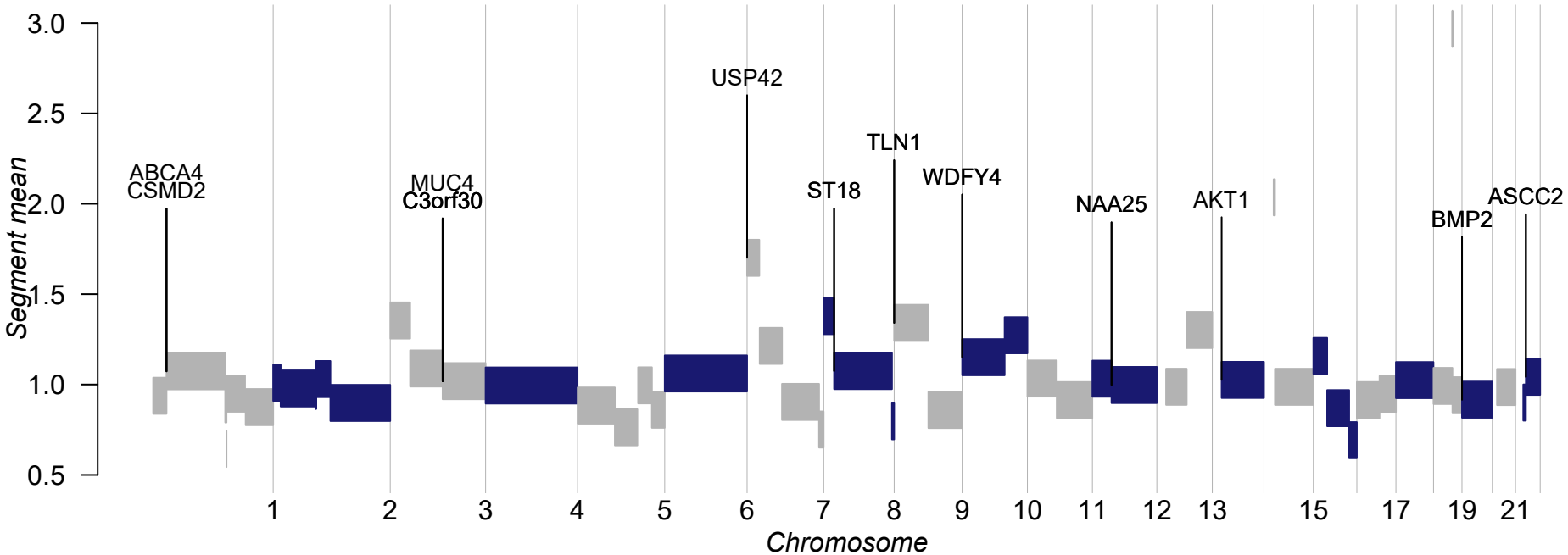

Supplement: Supplemental Information 3 [file peerj-13-19948-s003.zip › TK-CQ-2023-0609-004/3.CNV/cnvplot_zlyy001.pdf]

*zlyy002*

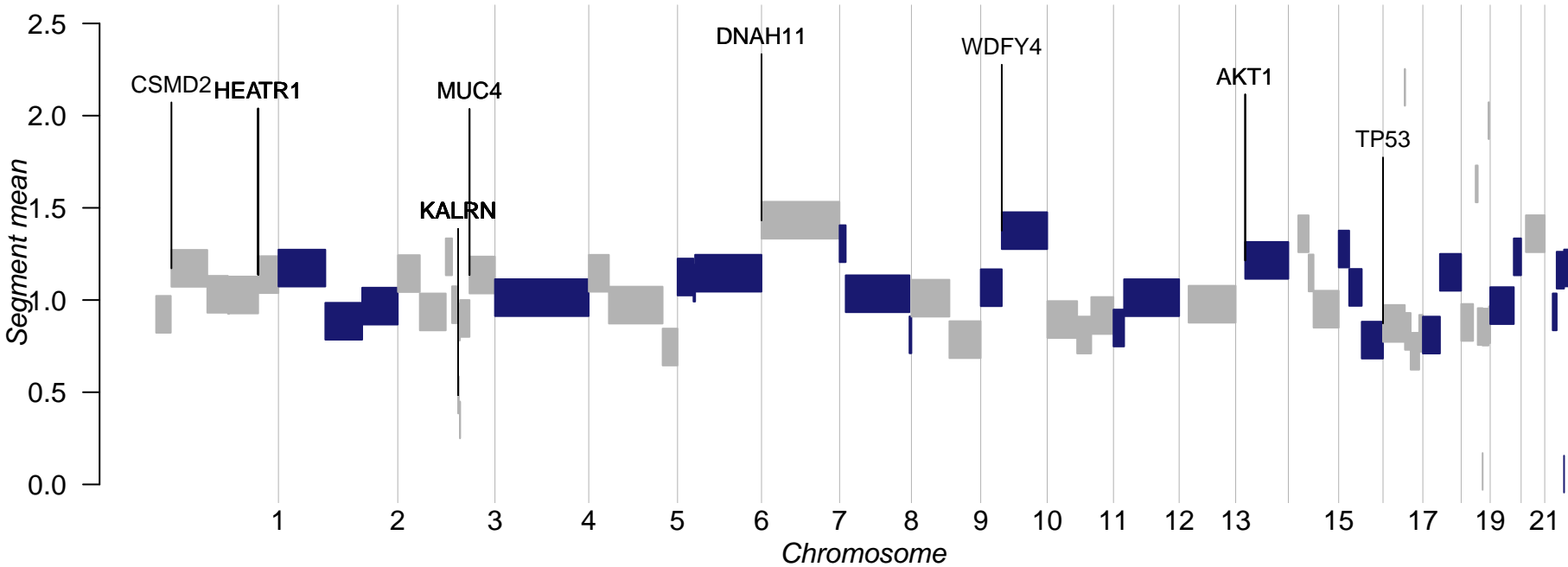

Supplement: Supplemental Information 3 [file peerj-13-19948-s003.zip › TK-CQ-2023-0609-004/3.CNV/cnvplot_zlyy002.pdf]

*zlyy003*

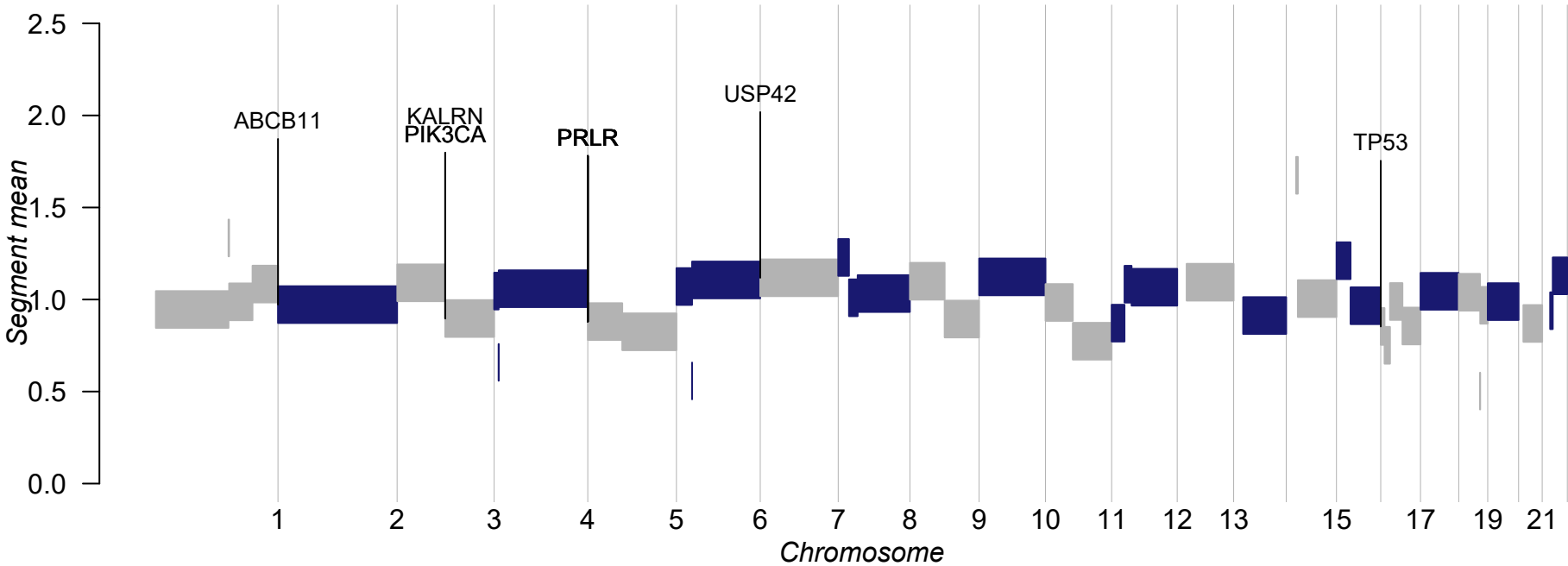

Supplement: Supplemental Information 3 [file peerj-13-19948-s003.zip › TK-CQ-2023-0609-004/3.CNV/cnvplot_zlyy003.pdf]
