# Supplementary material for: BMP2 alterations in mucinous cystadenocarcinoma of the breast: insights from whole-exome sequencing
Source: PeerJ. 2025 Sep 3;13:e19948. doi: 10.7717/peerj.19948 (PMC12422278; doi:10.7717/peerj.19948)
Supplement: Supplemental Information 3 [file peerj-13-19948-s003.zip › BMP2/BMP2/METABRIC BMP2 plot.pdf]

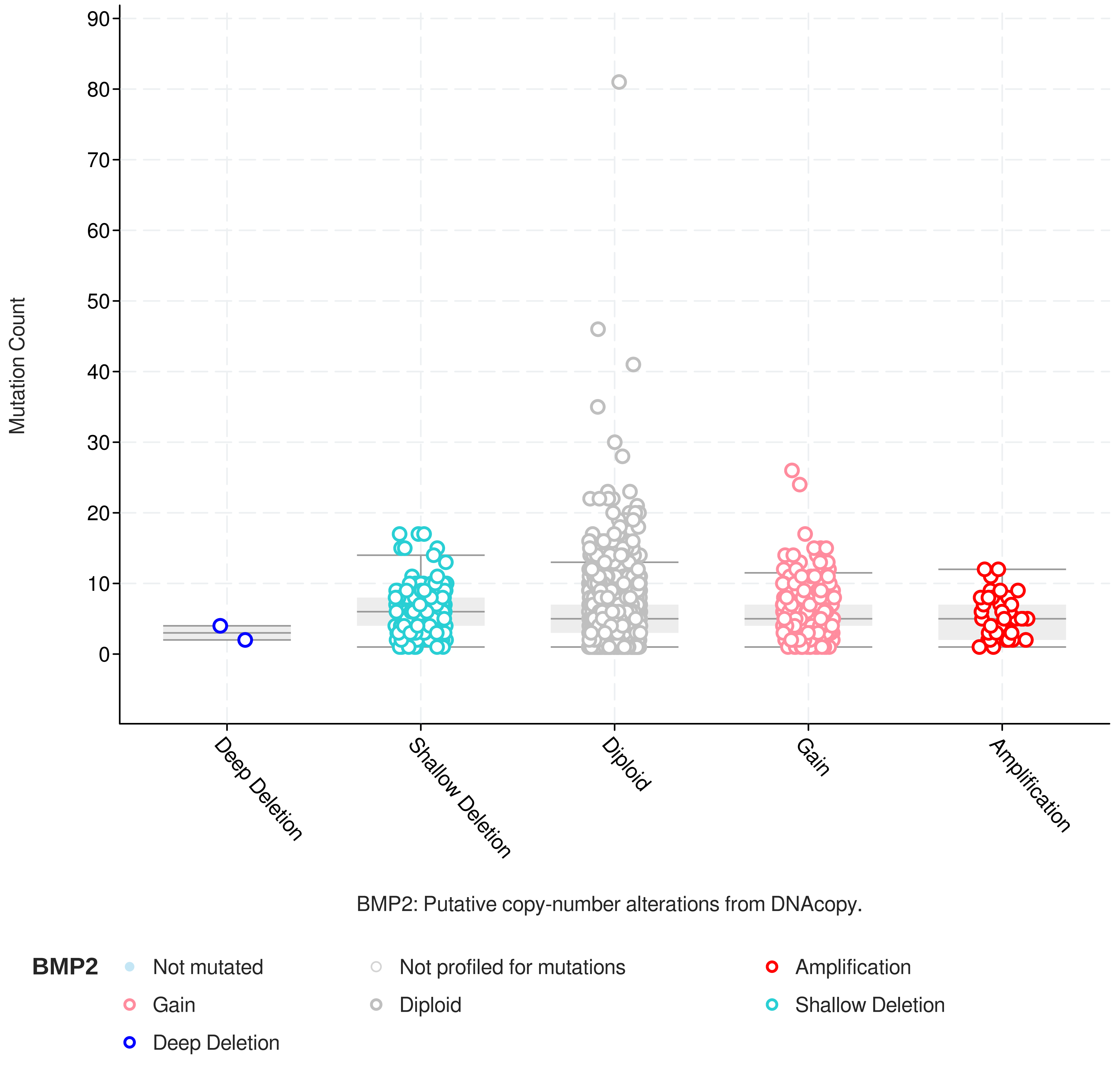

Mutation Count
90
80
70
60
50
40
30
20
10
0
Deep Deletion
Shallow Deletion
Diploid
Gain
Amplification
BMP2: Putative copy-number alterations from DNAcopy.
BMP2
Not mutated
Not profiled for mutations
Amplification
Gain
Diploid
Shallow Deletion
Deep Deletion
