# Supplementary material for: BMP2 alterations in mucinous cystadenocarcinoma of the breast: insights from whole-exome sequencing
Source: PeerJ. 2025 Sep 3;13:e19948. doi: 10.7717/peerj.19948 (PMC12422278; doi:10.7717/peerj.19948)
Supplement: Supplemental Information 3 [file peerj-13-19948-s003.zip › BMP2/BMP2/Scna.pdf]

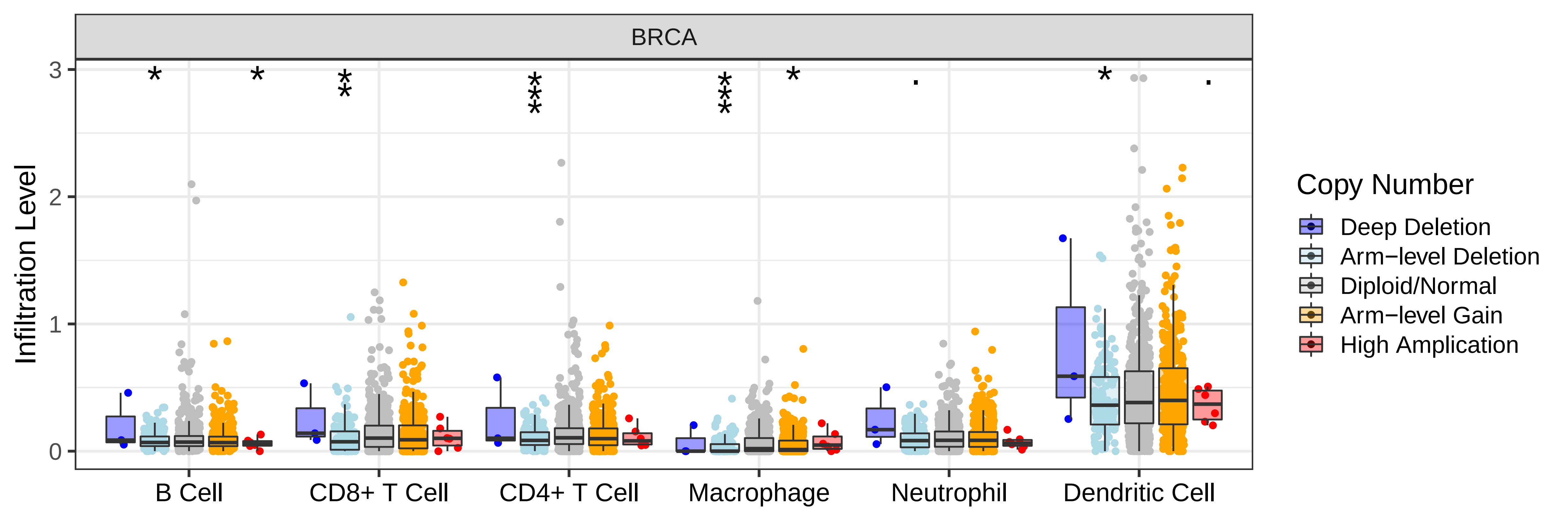

BRCA
Infiltration Level
3
2
1
0
*
*
**
***
***
*
.
*
.
B Cell
CD8+ T Cell
CD4+ T Cell
Macrophage
Neutrophil
Dendritic Cell
Copy Number
Deep Deletion
Arm−level Deletion
Diploid/Normal
Arm−level Gain
High Amplication
