# Supplementary material for: BMP2 alterations in mucinous cystadenocarcinoma of the breast: insights from whole-exome sequencing
Source: PeerJ. 2025 Sep 3;13:e19948. doi: 10.7717/peerj.19948 (PMC12422278; doi:10.7717/peerj.19948)
Supplement: Supplemental Information 5 [file peerj-13-19948-s005.docx]

The details regarding the WES assay in terms of sequencing information or variant calling information

Sequencing platform: DNBSEQ-T7

Library preparation reagents: Hieff NGS Ultima Pro DNA Library Prep Kit for Illumina SSELXT CRE V4

Sequencing specification: paired-end 150 bp depth >100x

Raw data filtering standard: Q30 ≥ 85%

fq quality control software: fastp, using the default parameters of the software

Comparison software: sentieon bwa mem uses the default parameters of the software

Deduplication method: gatk MarkDuplicates

Parameter：--OPTICAL_DUPLICATE_PIXEL_DISTANCE 2500 --ASSUME_SORT

_ORDER "coordinate" --CLEAR_DT false --CREATE_MD5_FILE true

Coefficient of variation：gatk parameter -T HaplotypeCaller -rf NotPrimaryAlignment -rf MaxInsertSize -maxInsert 1000 -rf BadCigar -rf BadMate --min_mapping

_quality_score 20 -rf MateSameStrand --min_base_quality_score 15

SNP parameter of filtration：QD < 2.0 || FS > 60.0 || MQ < 30.0, DP < 4

INDEL parameter of filtration：QD < 2.0 || FS > 200.0, DP < 4
