# Supplementary material for: BMP2 alterations in mucinous cystadenocarcinoma of the breast: insights from whole-exome sequencing
Source: PeerJ. 2025 Sep 3;13:e19948. doi: 10.7717/peerj.19948 (PMC12422278; doi:10.7717/peerj.19948)
Supplement: Supplemental Information 3 [file METABRIC BMP2 oncoprint.pdf › BMP2/BMP2/METABRIC BMP2 oncoprint.pdf]

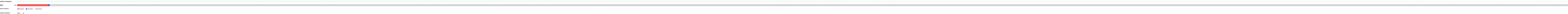
Profiled in Mutations
BMP2
2.1%
Genetic Alteration
Amplification
Deep Deletion
No alterations
Profiled in Mutations
Yes
No
