# Supplementary material for: BMP2 alterations in mucinous cystadenocarcinoma of the breast: insights from whole-exome sequencing
Source: PeerJ. 2025 Sep 3;13:e19948. doi: 10.7717/peerj.19948 (PMC12422278; doi:10.7717/peerj.19948)
Supplement: Supplemental Information 3 [file TCGA oncoprint BMP2.pdf › BMP2/BMP2/TCGA oncoprint BMP2.pdf]

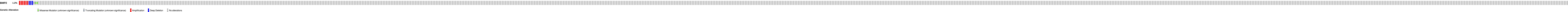
BMP2
1.2%
Genetic Alteration
Missense Mutation (unknown significance)
Truncating Mutation (unknown significance)
Amplification
Deep Deletion
No alterations
